# Combined Ligand/Structure-Based Virtual Screening and Molecular Dynamics Simulations of Steroidal Androgen Receptor Antagonists

**DOI:** 10.1155/2017/3572394

**Published:** 2017-02-15

**Authors:** Yuwei Wang, Rui Han, Huimin Zhang, Hongli Liu, Jiazhong Li, Huanxiang Liu, Paola Gramatica

**Affiliations:** ^1^School of Pharmacy, Lanzhou University, 199 West Donggang Rd., Lanzhou 730000, China; ^2^Department of Theoretical and Applied Sciences, University of Insubria, Via Dunant 3, 21100 Varese, Italy

## Abstract

The antiandrogens, such as bicalutamide, targeting the androgen receptor (AR), are the main endocrine therapies for prostate cancer (PCa). But as drug resistance to antiandrogens emerges in advanced PCa, there presents a high medical need for exploitation of novel AR antagonists. In this work, the relationships between the molecular structures and antiandrogenic activities of a series of 7*α*-substituted dihydrotestosterone derivatives were investigated. The proposed MLR model obtained high predictive ability. The thoroughly validated QSAR model was used to virtually screen new dihydrotestosterones derivatives taken from PubChem, resulting in the finding of novel compounds CID_70128824, CID_70127147, and CID_70126881, whose in silico bioactivities are much higher than the published best one, even higher than bicalutamide. In addition, molecular docking, molecular dynamics (MD) simulations, and MM/GBSA have been employed to analyze and compare the binding modes between the novel compounds and AR. Through the analysis of the binding free energy and residue energy decomposition, we concluded that the newly discovered chemicals can in silico bind to AR with similar position and mechanism to the reported active compound and the van der Waals interaction is the main driving force during the binding process.

## 1. Introduction

According to the latest World Cancer Report 2014 [1], prostate cancer (PCa) has become the second most common cancer among men in the world. The morbidity rate of PCa has reached 15%, which is merely 1.7% lower than the leading lung cancer. It is reported that about 1100,000 people were diagnosed as new PCa patients in 2012 [2]. Additionally researchers pointed out that prostate cancer is not the privilege of men; women have similar prostate tissue, which also has the risk of cancer [3].

The androgen receptor (AR), a ligand inducible transcription factor in the nuclear hormone receptor super family [4], plays a critical role in the development and progress of PCa. Natural hormone testosterone (T) and dihydrotestosterone (DHT), known as androgens, are the endogenous ligands of AR. When bound to AR, androgens play significant roles in the sexual development, function, and musculoskeletal growth of male. The main mechanism of androgen action is to regulate the gene expression by means of binding to AR, changing the level of specific proteins in cells, and controlling cell behavior [5]. Therefore, a rational approach to cure PCa is the use of antiandrogens to prevent the interaction of T or DHT with AR.

At present, androgen receptor antagonists, such as bicalutamide and flutamide, are used as main hormone therapies for prostate cancer [6]. Although these antiandrogens exhibit good efficacy in many cases and comprise an important part of effective therapeutics [7–10], the emergence of recurrent and metastatic forms of castration-resistant PCa (CRPC) becomes a major challenge, with a median survival of only 1~2 years [11]. A possible reason is that these antiandrogens have partial agonistic activities at high concentration in vitro [12]. Therefore, the discovery of new AR antagonists with high antiandrogen activities is highly expected.

Here, in this study, to aid the research and development of steroidal antiandrogens, we investigated the relationships between a series of 7*α*-substituted dihydrotestosterone derivatives and corresponding antiandrogen activities. The vital features related to the bioactivities were explored, and a linear quantitative structure-activity relationship (QSAR) model was established according to OECD principles [13], using the QSARINS program [14, 15]. Then the QSAR model, thoroughly and strictly validated by various internal and external validation techniques, is used to virtually screen new dihydrotestosterones, without experimental bioactivities, downloaded from PubChem database [16]. Besides, molecular docking and molecular dynamics (MD) simulations are used to study the possible binding mode of compounds owning high in silico activities with androgen receptor. At last, the most active compounds with good binding affinities to AR, as highlighted by the Insubria graph [17], are proposed for experimental research group to test the antiandrogen activities in the future.

## 2. Materials and Methods

### 2.1. Data Set

**Figure 1:**
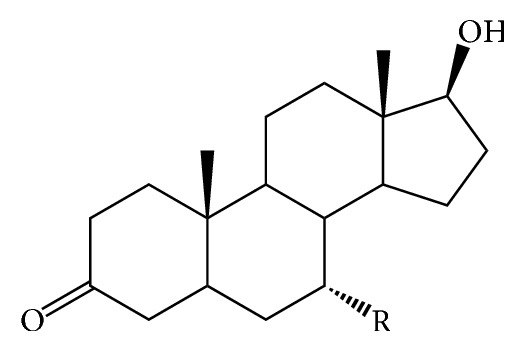
The skeleton structure of 7*α*-substituted dihydrotestosterones derivatives.

The success of any QSAR model depends on accurate and clean training data, proper representative descriptor selection methods, suitable statistical methods, and, most critically, both internal and external validation of resulting methods [18, 19]. Here, in this work, a set of 36 7*α*-substituted dihydrotestosterones derivatives were taken from literatures [20, 21]. The skeleton structure of these derivatives is shown in Figure 1, in which R group represents amine, carboxylic acids, and halogens, and so forth.

These molecules were divided into a training set and a prediction set according to the structure diversity in QSARINS. Finally, 29 compounds were included in the training set and 7 compounds were in the prediction set (prediction set a). The experimental values, half maximal inhibitory concentration (IC_50_) expressed in nM, were converted into negative logarithmic units marked as pIC_50_, which was used as dependent variables in the QSAR analyses. The studied molecular structures and corresponding antiandrogen activities were listed in Table 1.

### 2.2. Descriptors Calculation

To describe a molecule, the molecular structures were firstly sketched in HyperChem program [22] and then were optimized to the minimum energy conformation by using AM1 method. After minimization, we submit the structures to DRAGON 5.5 software [23] to calculate 2914 descriptors including zero-, one-, two-, and three-dimensional (0D, 1D, 2D, and 3D), charge descriptors, and molecular properties. The related theories of the molecular descriptors are provided by DRAGON software, and the calculation procedure is clarified in detail, in the Handbook of Molecular Descriptors [24].

In order to facilitate the successive feature selection process, the constant and near constant descriptors were removed. Besides, if pairwise correlation of two descriptors is larger than 0.98, the one showing the highest pairwise correlation with others will be excluded. Finally, 358 descriptors remained for the next variable selection process.

### 2.3. QSAR Model Generation

After descriptor calculation, genetic algorithm (GA) implemented in QSARINS software was used to select descriptors. The final model was built by using MLR method based on the selected descriptors, named GA-MLR. The first step of GA is to produce a set of solutions randomly which is called initial population. Each solution, a model based on the contained descriptors by using multiple linear regressions method, is called a chromosome. Subsequently, the fitness function, Friedman LOF, is used to evaluate the fitness of these individuals:

(1) $$\begin{array}{c} LOF={\left\{\;\frac{SSE}{\left[1-\left(c+dp/n\right)\right]}\;\right\}}^{2}. \end{array}$$

Here, SSE represents the sum of squares of errors, *c* is the number of basis function, *d* is the smoothness factor (default 0.5), *p* is the number of features in the model, and *n* is the number of samples for model construction. In the successful selection stage, the fittest individuals evolve to the next generation. Then crossover and mutation operators were performed to generate new individuals. Finally a new population is formed consisting of the fittest chromosomes. The above evolution continues until the stop conditions are satisfied. The related parameters that control the GA performance are list as follows: population size (200), maximum generations (10000), and mutation probability (0.05).

### 2.4. Model Validation

QSARINS is based on GA-MLR method and performed various tools to a rigorous internal and external validation, based on the different validation criteria, as well as for the check of model applicability domain.

The robustness and stability of the built model were validated by several statistical parameters, such as determination coefficient (*R*^2^), leave-one-out (LOO) cross-validation *Q*_LOO_^2^, and root mean squared error (RMSE). Besides, leave-many-out (LMO) cross-validation method was also performed and *Q*_LMO_^2^ was reported. Randomization technology, by reordering the independent variable, was used to exclude the possibility of the chance correlation. Generally, the correlation coefficient of the built QSAR model should exceed Y randomized generated model. After Y scrambling was carried out with iterations of 5,000, the average value of squared correlation coefficient of the randomized models *R*^2^ and *Q*_LOO_^2^ was reported.

A good QSAR model should also have satisfactory predictive ability. The best way to evaluate the predictive ability of a model is its validation by new compounds, called prediction set, which do not participate in the process of model building. After the activities of the prediction set samples were predicted, the agreement between the experimental and predicted values was calculated as a measure of a QSAR model quality. Here we adopt several ways to calculate this agreement, *Q*_*F*1_^2^, *Q*_*F*2_^2^ [25], *Q*_*F*3_^2^ [26], and CCC [27–29].

All the above external validation parameters were calculated in the software QSARINS and were combined to evaluate the predictive ability of the proposed model.

### 2.5. Applicability Domain

To validate the practical applicability of a model to a new chemical, the applicability domain (AD), a theoretical domain which is defined by means of the selected descriptors in the process of modeling, should be defined properly. In this research, the AD was quantitatively assessed by the leverage approach [30, 31]. The leverage (hat, *h*) was calculated by *h*_*i*_ = *x*_*i*_(*X*^*T*^*X*)*x*_*i*_^*T*^  (*i* = 1,…, *m*), where *x*_*i*_ was the descriptor row-vector of the query compound *i* and *X* was the *n∗p* matrix of the training set (*p* is the number of model descriptors). The limit of model domain was quantitatively defined by the leverage cutoff (*h*^*∗*^), set as 3(*p* + 1)/*n*. A leverage greater than *h*^*∗*^ means that the query was outside of the model structural AD, so the predictions were extrapolations of the model and could be less reliable. The AD for chemicals with experimental data was verified by the Williams plot, where the hat values versus the standardized residuals were plotted, while the AD for chemicals without experimental data, which were analyzed in the virtual screening, was verified by the Insubria graph where the hat values were plotted versus the predicted responses [14, 18].

### 2.6. Virtual Screening of Potent Steroidal Antiandrogens

To explore more 7*α*-substituted dihydrotestosterones and to find similar derivatives with high antiandrogen activities, the studied skeleton structure was used as a query to search PubChem database for new dihydrotestosterones, without experimental bioactivities. Then the established MLR model, after thoroughly being validated internally and externally, was used to predict the antiandrogenic activities of these new dihydrotestosterones, verifying the AD.

Besides, molecular docking was employed to investigate the possible interaction mechanisms of the samples owning high in silico antiandrogenic activities with AR. Particularly, comprehensively considering the docking speed and accuracy, LigandFit, which is commonly used as a flexible docking method executed in the commercial software Discovery Studio 2.5 [32], was applied into the progress of structure-based virtual screening. The protein structure of AR was firstly downloaded from RCSB Protein Data Bank [33] (PDB entry code: 1T65) and imported in docking process. All ligands and water molecules were removed at first, the charge and polar hydrogen atoms were added, and the incomplete residues were corrected.

### 2.7. Molecular Dynamics (MD) Simulations

The molecular dynamics (MD) simulations were carried out using the Amber 14 software package [34]. MD is a commonly used methodology in exploring the interaction between ligand and protein. We have investigated the interaction mechanisms of R-bicalutamide/S-1 with WT/W741L AR using molecular dynamics simulations [35]. The docked structures of AR (PDB ID: 1T65) with the reported most active compound number 4 and novel chemicals with high in silico activities were used as the initial structures for MD simulations. During the process of docking, taking into consideration the fact that these residues collide significantly with the compounds, Helix 12 of AR was removed in the model as executed in the literature [36].

All missing hydrogen atoms of the AR were added by the LEaP module of the Amber 14 package. To maintain the electroneutrality of all the studied complexes, the appropriate number of chloride counterions was added. Then each complex was immersed into a cubic periodic box of TIP3P water model [37] with at least 10 Å distance around the complex.

For the ligand, the GAFF parameter assignments [38] were made by using Antechamber program and the partial charges were assigned by using the AM1-BCC method [39].

Amber 14 package and the Amberff03 force field were used for all molecular dynamics simulations. Sander program was carried out for the energy minimization and equilibration protocol. First, energy minimization of four complexes was done through three stages, using the steepest descent method switched to a conjugate gradient every 2500 steps for a total of 5000 steps with a nonbonded cutoff of 10 Å. In the first stage, to enable the added TIP3P water molecules to adjust to their proper orientations, the AR and ligand were restrained with 5.0 kcal mol^−1^ Å^−2^. In the second stage, to enable the AR to find a better way of accommodating ligand, the protein backbone was restrained with 3.0 kcal mol^−1^ Å^−2^. In the third stage, the entire solvated system was minimized without any restraint. Additionally, gradual heating, density, and equilibration protocols were performed. During the 100 ps heating procedure, the system was gradually heated from 0 to 310 K, and then the density was at 310 K for 400 ps, and at last the equilibration was at 310 K for 400 ps.

Afterwards, four 20 ns production MD simulations were carried out with the PMEMD program without any restraints in the isothermal isobaric ensemble (NPT, *P* = 1 atm, *T* = 310 K) MD. The time step was set at 2 fs. 10 Å cutoff was applied to treat nonbonding interactions. During the simulations periodic boundary conditions were employed and all electrostatic interactions were calculated using the particle mesh Ewald (PME) method. The SHAKE algorithm was used to restrain all bond lengths containing hydrogen atoms. All of the coordinate trajectories were recorded every 2 ps throughout all MD runs. To analyze the energy and structure, a total of 500 snapshots of the simulated structures stripped in the last 2 ns stable MD production trajectory at 4 ps intervals were extracted.

### 2.8. Binding Free Energy Calculations

For each protein-ligand complex, the binding free energy was analyzed by the MM/GBSA method [40]. To compare the AR binding free energies with different ligands, MM/GBSA calculation was applied to the 500 snapshots extracted from the final 2 ns of the MD trajectories. The total free energy of binding free energy was composed of the following molecular species (complex):

(2) ΔGbindGcomplex−Gprotein−Gligand=ΔEMM+ΔGsol−TΔS,

where *G*_complex_, *G*_protein_, and *G*_ligand_ are the free energy of complex, receptor, and ligand, respectively. The free energy for each species (complex, ligand, or receptor) can be decomposed into a gas phase energy (Δ*E*_MM_), a solvation-free energy (Δ*G*_sol_), and an entropy term (*T*Δ*S*).

(3) ΔEMM=ΔEval+ΔEele+ΔEvdw,ΔGsol=ΔGp+ΔGnp,ΔGnp=γSASA+β,

where the Δ*E*_MM_ is the sum of the internal energy of bonds, angle, and torsion (Δ*E*_val_), electrostatic interaction energy (Δ*E*_ele_), and van der Waals interaction energy (Δ*E*_vdw_). Δ*G*_sol_ is solvation-free energy and can be divided into two parts, the polar solvation-free energy (Δ*G*_p_) and the nonpolar solvation-free energy (Δ*G*_np_). The polar solvation-free energy Δ*G*_p_ is determined by generalized Born (GB) equation. The values of the dielectric constant for solute and solvent were set as 1 and 80. Δ*G*_np_ is the nonpolar solvation contribution and was calculated with constants 0.0072 kcal mol^−1^ Å^−2^ for surface tension proportionality constant *γ* and 0.92 kcal mol^−1^ Å^−2^ for the nonpolar free energy for a point solute *β*. SASA is the solvent accessible surface area and is determined by recursively approximating a sphere around each atom, starting from icosahedra (ICOSA method). *T*Δ*S* is the entropy term, including the translational, rotational, and vibrational terms of the solute molecules.

### 2.9. Energy Decomposition

Furthermore, to obtain the contribution of each residue to the binding process, we performed binding free energy decomposition. The MM/GBSA approach was used to calculate the per-residue free energy decomposition, which is based on the same 500 snapshots we have extracted from the last 2 ns of the stable MD trajectory.

### 2.10. Normal Mode Calculation

Entropy was analyzed by normal mode with AMBER14 NMODE module. Due to the high computational cost in the entropy calculation, 50 snapshots were extracted from the last 2 ns trajectory of the simulation with 40 ps time intervals.

## 3. Results and Discussion

### 3.1. The Linear MLR Model

The training set samples, 29 compounds as listed in Table 1, were used to build QSAR model by using GA-MLR methods, and the remaining compounds were used to evaluate the predictive ability of the built model. GA provided a series of linear equations containing different descriptor combinations with different performance, but similarly satisfactory. An excellent QSAR model should have high fitting ability, high cross-validated *Q*_LOO_^2^, high external predictive ability, and little difference between internal and external predictive ability.

Based on the above principles, a four-descriptor model was selected as the final model. The involved descriptors and corresponding physical-chemical meanings were listed in Table 2. The corresponding model equation and statistic parameters are listed as follows:

(4) pIC50=−2.89IC5+1.01GATS5e−3.17DISPp−12.99HATS3u+27.13,R2=0.760,QLOO2=0.656,QLMO2=0.662,QF12=0.739,QF22=0.731,QF32=0.876,CCC=0.891,RMSEtraining=0.226,RMSEprediction=0.270.

From the linear equation and statistic parameters, we could see that the fitting ability of the final model was relatively high with *R*^2^ of 0.760 and the final model was stable with *Q*_LOO_^2^ of 0.656 and *Q*_LMO_^2^ of 0.662. About the predictive ability of the final model, we could find that *Q*_*F*1_^2^ and *Q*_*F*2_^2^ have similar high values. Compared with *Q*_*F*1_^2^ and *Q*_*F*2_^2^, the value of *Q*_*F*3_^2^ was higher. Besides, the value of CCC was as high as 0.891, surpassing the threshold value of 0.85 as suggested in literature [29] for predictive model. Additionally, the RMSE values for the training set and prediction set were similarly very low. All these parameters indicated the higher external prediction ability of the final model. The interrelation coefficients of the selected descriptors were presented in the Table 3. It could be seen that the highest intercorrelation coefficient *K* was −0.61 between IC5 and HATS3e, which indicated that the used variables were independent. All results proved that the selected model was reliable, stable, and predictive.

**Figure 2:**
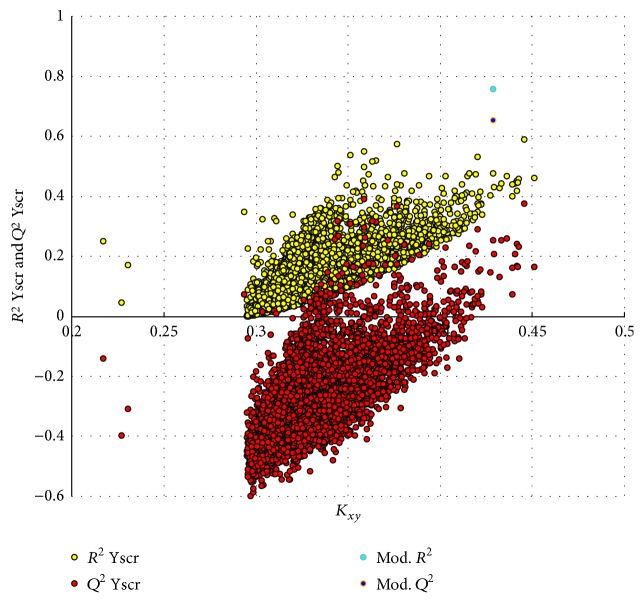
The distribution of *R*^2^ and *Q*^2^ of 5000 iterated Y-scrambled models in comparison to the proposed model performances.

Y randomization technique was carried out with iterations of 5000 in QSARINS.  Figure 2 showed the plot of *R*^2^ and *Q*^2^ values versus *K*_*xy*_, automatically obtained in QSARINS. From Figure 2, we could find that the *R*^2^ and *Q*^2^ values of the final model were much higher than the models from scrambled Y-column, because the relationship between molecular structure and response was broken. This result indicated that the relationships between structures of 7*α*-substituted dihydrotestosterones and corresponding pIC_50_ values did exist in the proposed model, and it was really not obtained by chance.

**Figure 3:**
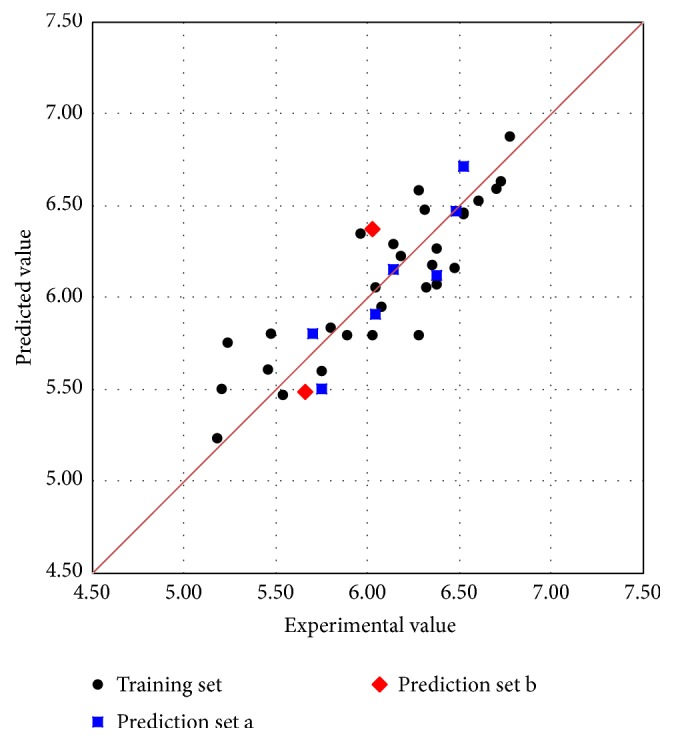
The scatter plot of the experimental versus the predicted pIC_50_ by MLR model.

The predicted pIC_50_ values by MLR model were listed in Table 1.  Figure 3 was the scatter plot of the experimental versus the predicted pIC_50_ values. It was obvious that, in Figure 3, all predicted pIC_50_ values were close to the line *y* = *x*, which indicated that the linear model can accurately predict the antiandrogenic values of these derivatives.

**Figure 4:**
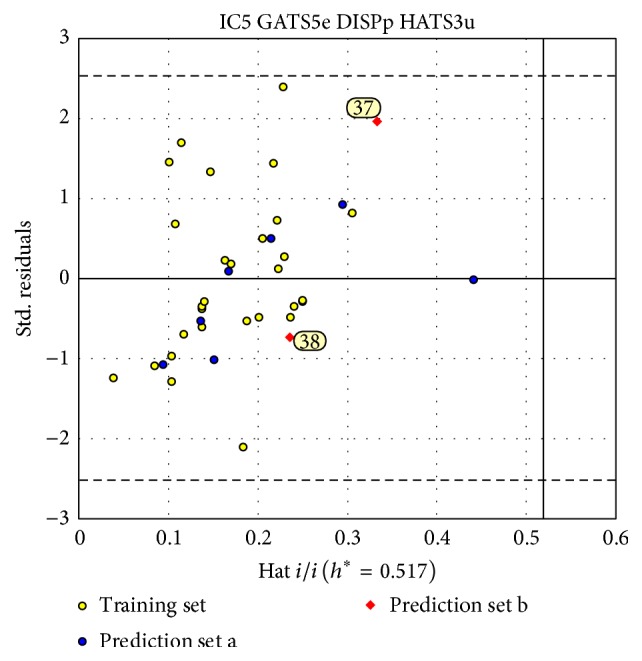
The Williams plot of final MLR model.

The model applicability domain was evaluated by means of leverage analysis, namely, Williams plot, shown in Figure 4, in which the standardized residuals (*σ*) and leverage values (*h*) were plotted. In Figure 4, we could see that all compounds were inside the model structural applicability domain (*h*^*∗*^ = 0.517) and reasonably well predicted with standard residue smaller than 2.5*σ*.

After the MLR model was built, we luckily found two new 7*α*-substituted dihydrotestosterones, showed in Table 1 (marked as “b,” prediction set b), with experimental antiandrogen activities from another published literature [41]. These two compounds were additionally used to validate our model. Both of them (numbered in the Williams plot of Figure 4) were located in the applicability domain of the MLR model with *h* value of 0.332 for compound 37 and 0.237 for compound 38. The predictions on them were quite near to their experimental values. In Figure 3, these two samples (in red) were very near to the line *y* = *x*. These results further indicated the high predictive ability of the proposed MLR model.

By interpreting the meaning of the descriptors used in the model, we could extract vital structural features, to some extent, responsible for the antiandrogenic activities of these steroidal derivatives. IC5 was calculated as the mean information content as follows: IC5 = −∑_*g*−1_^*A*_*g*_^(*A*_*g*_/nAT) · log_2_(*A*_*g*_/nAT), where *g* runs over the equivalence classes, *A*_*g*_ was the cardinality of the *g*th equivalence class, and nAT was the total number of atoms. This index represented a measure of structural complexity per vertex. GATS5e belonged to 2D autocorrelations and was Geary autocorrelation, lag 5/weighted by atomic Sanderson electronegativities. This descriptor was favorable to the antiandrogen activities of these steroidal derivatives. DISPp, geometrical descriptors, indicated the displacement between the geometric center and the center of the polarizability, calculated with respect to the molecular principal axes. HATS3u is a GETAWAY descriptor [42], representing the leverage-weighted autocorrelation of lag 5/weighted by atomic polarizabilities. With the increase of these two descriptors, the bioactivities of the studied compounds decreased.

### 3.2. Virtual Screening

**Figure 5:**
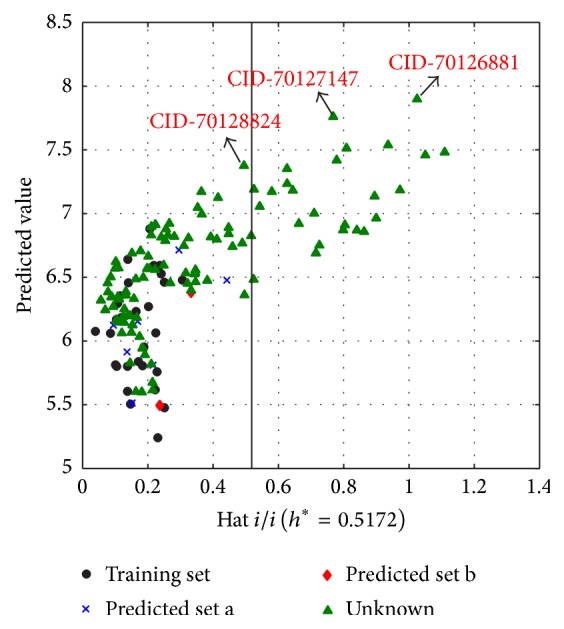
Insubria graph (plot of hat values versus predicted values for the complete compounds).

From PubChem database, we found 110 new 7*α*-substituted dihydrotestosterone derivatives, without experimental data. By exploring the leverage *h* values, 77.27% of them were located in the structural applicability domain of the proposed MLR model.  Figure 5 is the Insubria graph of these dihydrotestosterones, the plot of leverage values versus predicted pIC_50_, which was proposed especially for exploring the unknown samples. In Figure 5, most chemicals are in the range of the hat cutoff (*h*^*∗*^ = 0.517). Inside the model AD, the most active compound is CID_70128824, which has in silico PIC_50_ of 7.37, higher than the reported most active compound number 4 (pIC_50_ = 6.77). Outside the model AD, we luckily obtained several samples with higher in silico activities, especially CID_70126881 and CID_70127147, which showed excellent in silico antiandrogen activities as high as 7.90 and 7.76, respectively, even higher than bicalutamide and hydroxyflutamide. The ID of these compounds and corresponding predicted pIC_50_ values are listed in Table 4. Though only these three compounds were highlighted here, other samples with high in silico activities, especially those located in the model structural AD, were also worthy of our attention.

To further explore the possible binding mode of the screened compounds, molecular docking was employed to study the interaction between compounds owning high in silico activities (especially CID_70128824, CID_70126881, and CID_70127147), together with the reported most active compound 4 as a comparison, and androgen receptor (PDB ID: 1T65) by using LigandFit module in Discovery Studio 2.5. Firstly, DHT was extracted from crystal structure and redocked into ligand binding pocket to obtain the optimal docking parameters. Secondly, the ligand binding site was defined with the same parameters as DHT. At this point, the radius of SBD_Site_Sphere was set to 10 Å. The other parameters were set by default.

**Figure 6:**
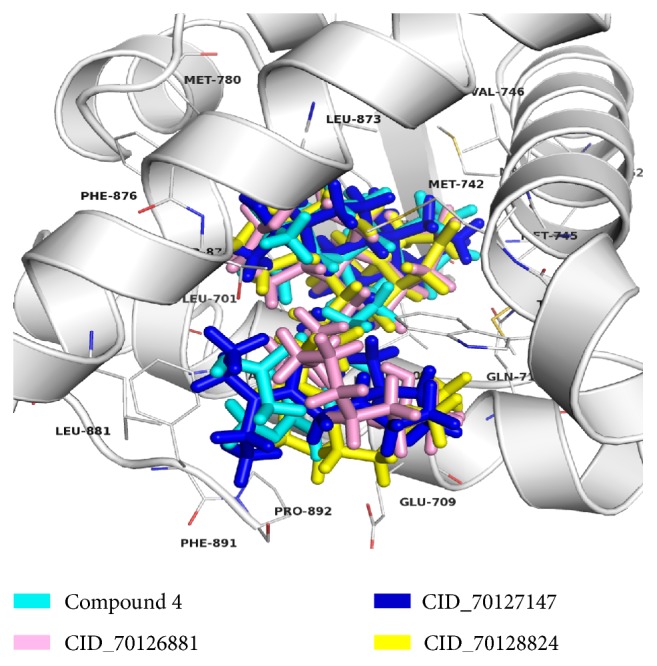
The molecular binding models of compound 4 (cyan), CID_70128824 (yellow), CID_70126881 (pink), and CID_70127147 (blue) in the AR ligand binding site.

To obtain reasonable conformations of different complex, the top-ranked compounds with lowest RMSD values were extracted. The binding mode of compound 4, CID_70128824, CID_70126881, and CID_70127147 with AR were presented in Figure 6. From this Figure, it could be seen that the docked pose of CID_70128824, CID_70126881, and CID_70127147 located in the same position with similar orientation in the AR ligand binding site to compound 4. All these results indicated that though two of them were outside of the model AD, these three compounds CID_70128824, CID_70126881, and CID_70127147 might have good performance to antagonize androgen receptor and have the potency for further research and development for PCa therapy.

### 3.3. MD Simulations

#### 3.3.1. System Equilibration

**Figure 7:**
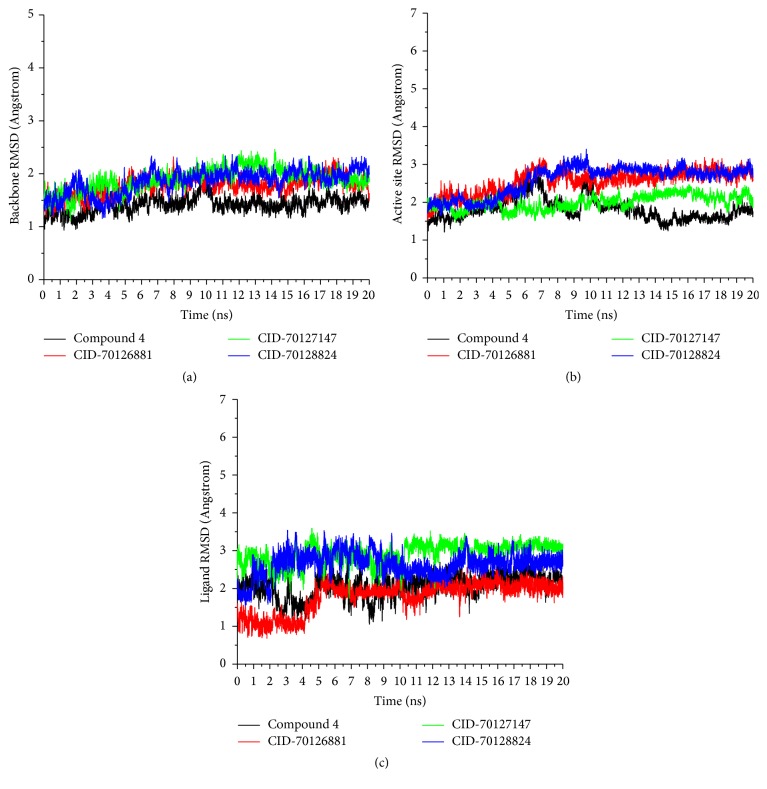
Time series of (a) the RMSDs of backbone atoms of androgen receptor, (b) the RMSD of C_*α*_ atoms for the residues around 5 Å of the ligand, and (c) the RMSD of the heavy atoms of ligand.

In order to verify whether the studied systems reach equilibrium, the root mean square deviations (RMSDs) of all the backbone atoms of the protein, the C_*α*_ atoms for the residues of the active site (residues within 5 Å around ligand), and the heavy atoms of ligand from the initial structure were monitored to examine the dynamic stability of the systems and plotted against time, as shown in Figure 7. The three RMSDs have small fluctuations after 15 ns, implying that the studied systems have reached stability. We used the last 2 ns to analyze the energy and binding modes for the four complexes.

#### 3.3.2. Validation of the MD Simulations

We calculated the binding free energy by MM/GBSA method between the four ligands and AR to validate the reliability of the MD simulation.  Table 5 lists the binding free energy and all of the energy terms for the four compounds. From Table 5, the four complexes had different binding free energy; the ranking order is CID_70126881 (−41.62 kcal mol^−1^), CID_70127147 (−33.06 kcal mol^−1^), CID_70128824 (−31.86 kcal mol^−1^), and compound 4 (−22.69 kcal mol^−1^). Different binding free energy means different binding affinity between the four complexes. We have proved that the antiandrogen activity of compounds CID_70126881, CID_70127147, and CID_70128824 are higher than the published best one (compound 4) by MLR model. In addition, the ranking of the calculated binding free energy was consistent with their in silico bioactivities order.

#### 3.3.3. Analysis of the Interaction Mechanism

According to the calculated binding free energy, CID_70126881 holds the strongest binding affinity; on the contrary, compound 4 has the lowest binding affinity. As can be seen from Table 5, the nonpolar interactions (Δ*G*_nonpolar_) including van der Waals (*E*_vdw_) and nonpolar solvation (Δ*G*_np_) terms are the driving force for the binding of the four ligands to AR, and the total polar contributions (Δ*G*_p_) are unfavorable for their binding. In addition, CID_70126881, CID_70127147, and CID_70128824 have almost the same van der Waals interactions toward AR (−69.32 kcal mol^−1^, −71.34 kcal mol^−1^, and −70.56 kcal mol^−1^ for CID_70126881-AR, CID_70127147-AR, and CID_70128824-AR), while compound 4 has a low van der Waals value, which may partly explain the reduced binding affinity of compound 4 and prove that the newly discovered chemicals could possess higher antiandrogen activities.

**Figure 8:**
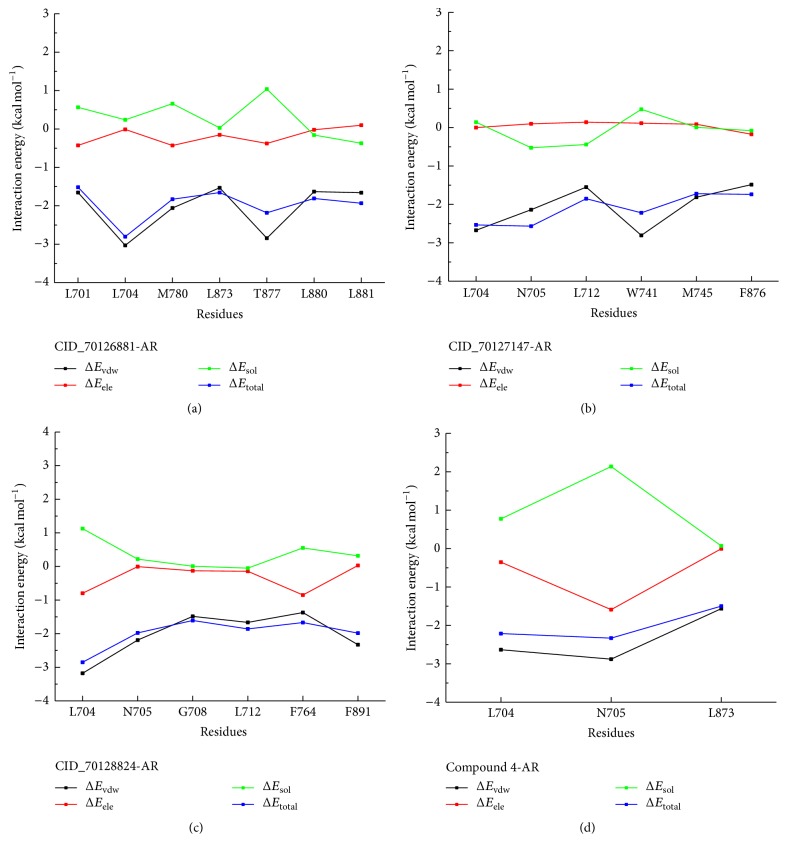
Energy decomposition of key residues in four complexes.

To obtain the detailed interaction between four ligands and AR, the decomposition of binding free energy, which is calculated by MM/GBSA method, was executed to identify key residues during the binding process. The result of energy decomposition contains van der Waals, electrostatic, solvation-free energy, and total energy contribution terms, respectively, for four systems, shown in Figure 8. All the residues with great energy contributions were almost more than 1.5 kcal mol^−1^. As shown in Figure 8(a), residues L701, L704, M780, L873, T877, L880, and L881 of AR make a significant contribution to the CID_70126881-AR binding, as well as those for the CID_70127147-AR which are L704, N705, L712, W741, M745, and F876 (Figure 8(b)). In Figure 8(c), residues L704, N705, G708, L712, F764, and F891 of AR make a substantial contribution to the CID_70128824-AR binding. However, only two key residues (L704 and N705) were the major energy contributions to compound 4-AR binding as shown in Figure 8(d). As mentioned previously, the vast majority of key residues of AR were nonpolar; it was reasonable to speculate that these residues can form greater van der Waals interactions with hydrophobic ligand and exhibit more favorable nonpolar interaction contribution to the binding free energy.

The MD simulation, together with the docking results, confirmed that the newly discovered chemicals CID_70126881, CID_70127147, and CID_70128824 share similar binding mode with the reported compound 4, and the in silico antiandrogen activities of them are higher through the calculated binding free energy and decomposition of binding free energy.

## 4. Conclusions

In this study, the relationships between a series of 7*α*-substituted dihydrotestosterone derivatives and corresponding antiandrogen activities were explored. A reliable, stable, and robust linear MLR model with four descriptors was built and validated in QSARINS. The predictive ability of the final model, fully evaluated by using two different prediction sets, is excellent enough to be used to virtually screen novel 7*α*-substituted dihydrotestosterones from PubChem database. After antiandrogenic activity prediction, molecular docking, and molecular dynamic simulations, CID_70126881, CID_70127147, and CID_70128824, as the most potent chemicals with good binding affinities to androgen receptor, were proposed. Of course, bioassay experimental researches are needed to evaluate the virtual screening results. This study provides the theoretical basis and specific chemicals for AR antagonists, which can help the experimental research groups to search for potential antiandrogens.

## Figures and Tables

**Table 1:** The studied compounds and corresponding experimental and predicted pIC_50_ values.

Number	Structure	Experimental pIC_50_	Predicted pIC_50_
1^a^	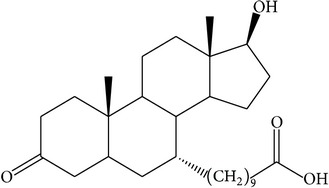	6.04	5.91
2^a^	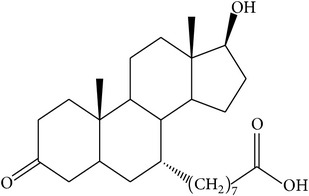	6.14	6.15
3^a^	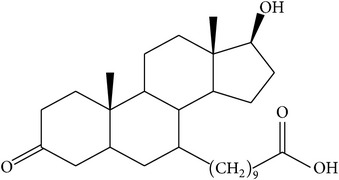	5.70	5.81
4	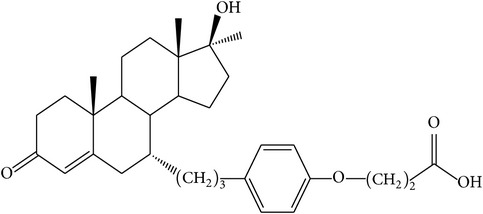	6.77	6.88
5	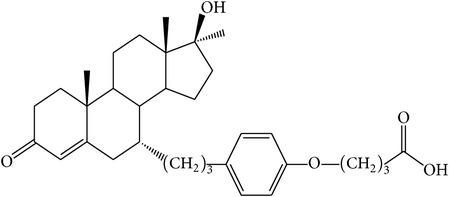	6.18	6.23
6^a^	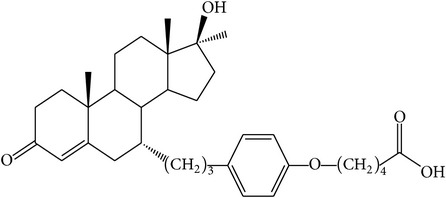	6.52	6.71
7	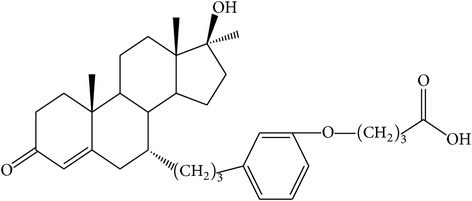	5.24	6.76
8	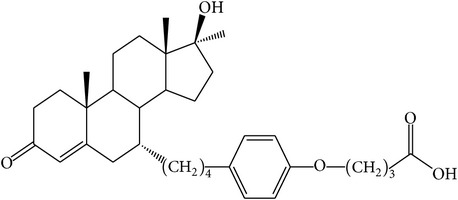	6.07	5.95
9	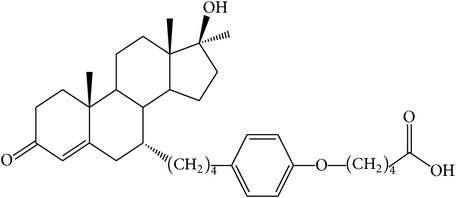	6.60	6.53
10	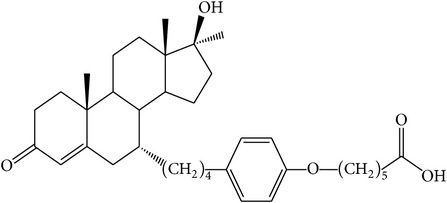	6.38	6.27
11	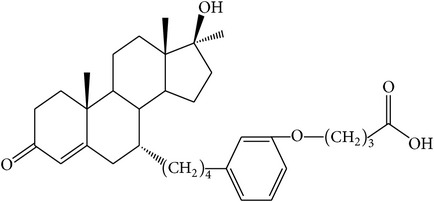	6.04	6.06
12^a^	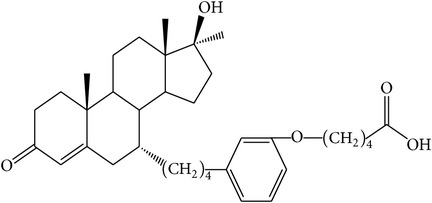	6.48	6.48
13	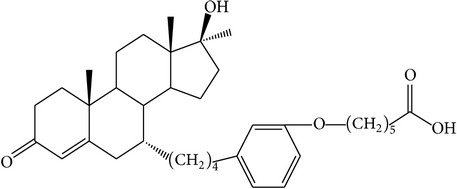	6.28	5.80
14	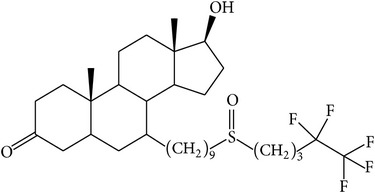	5.54	5.47
15	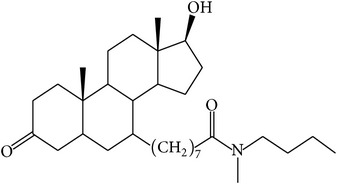	6.47	6.17
16	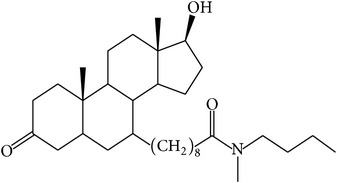	5.47	5.81
17^a^	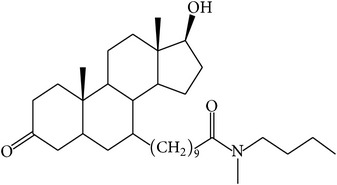	5.74	5.51
18	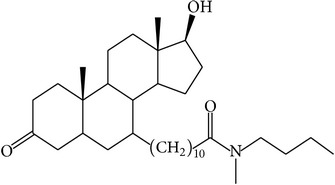	5.89	5.80
19	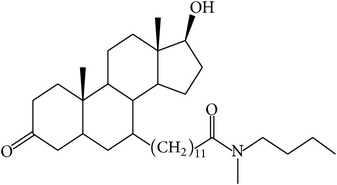	5.46	5.61
20	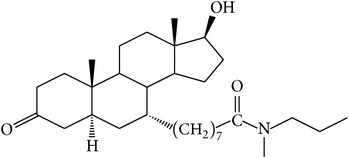	5.96	6.35
21^a^	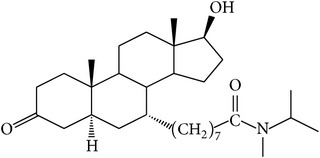	6.38	6.12
22	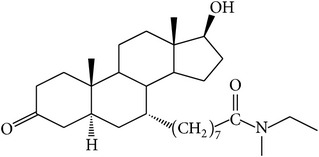	6.38	6.07
23	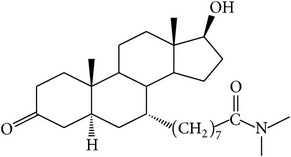	6.72	6.64
24	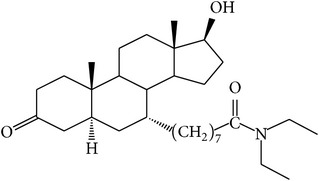	6.52	6.46
25	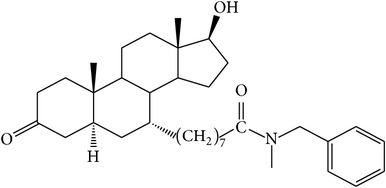	5.80	5.84
26	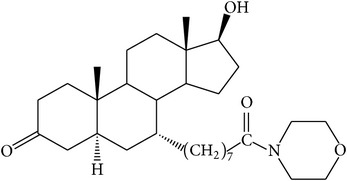	6.14	6.30
27	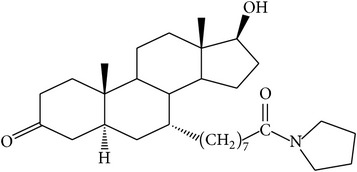	6.52	6.46
28	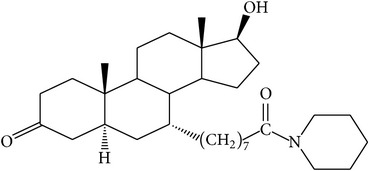	6.31	6.48
29	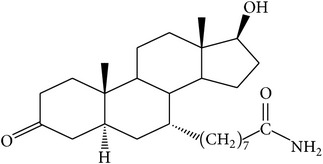	6.35	6.18
30	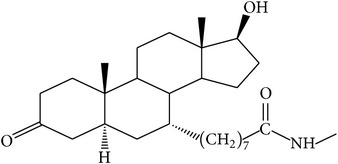	6.32	6.06
31	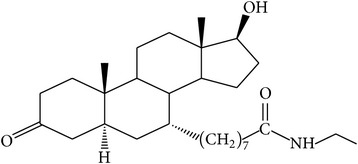	6.03	5.80
32	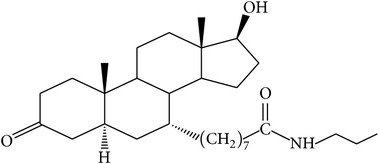	5.74	5.60
33	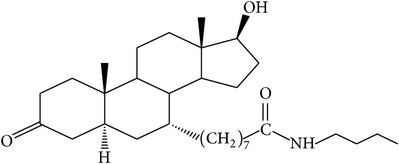	5.20	5.50
34	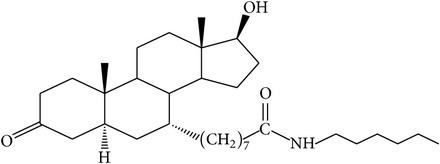	5.18	5.24
35	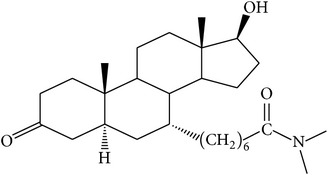	6.70	6.59
36	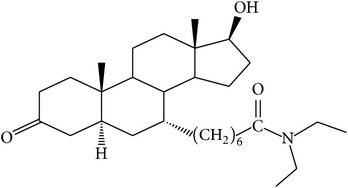	6.28	6.59
37^b^	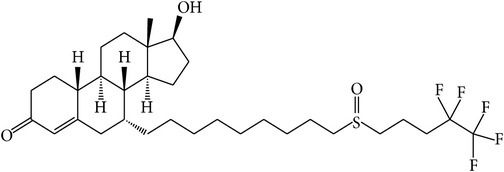	6.02	6.38
38^b^	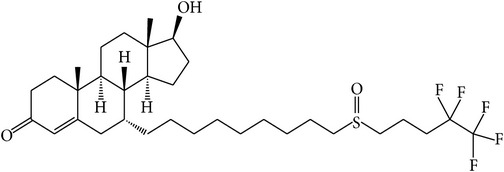	5.66	5.49

^a^The prediction set a; ^b^the prediction set b (Bradbury et al., 2011).

**Table 2:** The selected descriptors used to build QSAR model and corresponding meanings.

Descriptor	Meaning	Descriptor type
IC5	Information content index (neighborhood symmetry of 5-order)	Information indices
GATS5e	Geary autocorrelation, lag 5/weighted by atomic Sanderson electronegativities	2D autocorrelations
DISPp	d COMMA2 value/weighted by atomic polarizabilities	Geometrical descriptors
HATS3u	Leverage-weighted autocorrelation of lag 3/unweighted	GETAWAY descriptors

**Table 3:** The correlation coefficients (*K*) of the selected descriptors in the model.

	IC5	GATS5e	DISPp	HATS3u
IC5	1			
GATS5e	0.07	1		
DISPp	0.25	0.36	1	
HATS3u	−0.625	0.19	−0.15	1

**Table 4:** The 110 new compounds from PubChem database and corresponding predicted activities.

Number	MolID	Pred	AD^a^
1	CID_44421999	6.32	Y
2	CID_44422008	6.49	Y
3	CID_44422014	6.53	Y
4	CID_44422020	6.47	Y
5	CID_44422031	6.34	Y
6	CID_44422034	6.15	Y
7	CID_44422037	6.14	Y
8	CID_44422041	6.07	Y
9	CID_44422043	5.82	Y
10	CID_44422044	5.67	Y
11	CID_44422045	5.62	Y
12	CID_44422047	6.69	N
13	CID_44422053	6.16	Y
14	CID_44422054	6.91	Y
15	CID_44422058	6.90	Y
16	CID_44422064	6.12	Y
17	CID_44422067	5.94	Y
18	CID_44422075	6.25	Y
19	CID_44422080	6.33	Y
20	CID_44433644	6.53	Y
21	CID_44433645	6.20	Y
22	CID_67854257	7.51	N
23	CID_69758112	6.69	Y
24	CID_70126216	7.35	N
25	CID_70126231	7.17	Y
26	CID_70126247	6.91	Y
27	CID_70126297	5.60	Y
28	CID_70126298	5.83	Y
29	CID_70126305	6.56	Y
30	CID_70126327	7.42	N
31	CID_70126491	6.40	Y
32	CID_70126782	6.27	Y
33	CID_70126784	6.24	Y
34	CID_70126798	6.48	N
35	CID_70126802	6.87	N
36	CID_70126837	6.36	Y
37	CID_70126868	6.75	N
38	CID_70126881	7.90	N
39	CID_70126979	6.39	Y
40	CID_70126991	6.89	Y
41	CID_70127144	6.82	Y
42	CID_70127147	7.76	N
43	CID_70127181	6.03	Y
44	CID_70127183	6.35	Y
45	CID_70127185	6.87	N
46	CID_70127188	6.96	N
47	CID_70127192	6.88	Y
48	CID_70127194	6.81	Y
49	CID_70127269	6.35	Y
50	CID_70127287	7.48	N
51	CID_70127296	6.92	N
52	CID_70127297	6.77	Y
53	CID_70127298	6.82	Y
54	CID_70127444	6.80	Y
55	CID_70127446	7.54	N
56	CID_70127567	5.89	Y
57	CID_70127714	7.00	N
58	CID_70127721	6.57	Y
59	CID_70127722	6.82	Y
60	CID_70127760	6.91	N
61	CID_70127771	6.66	Y
62	CID_70127818	7.06	N
63	CID_70127821	7.17	N
64	CID_70128062	6.59	Y
65	CID_70128068	7.19	N
66	CID_70128078	6.06	Y
67	CID_70128083	6.83	Y
68	CID_70128084	6.81	Y
69	CID_70128209	6.75	Y
70	CID_70128238	6.83	Y
71	CID_70128450	6.33	Y
72	CID_70128452	7.23	N
73	CID_70128456	6.71	Y
74	CID_70128462	6.84	Y
75	CID_70128533	6.79	Y
76	CID_70128534	6.92	Y
77	CID_70128574	7.18	N
78	CID_70128608	7.18	N
79	CID_70128824	7.37	Y
80	CID_70128828	6.46	Y
81	CID_70128830	7.00	Y
82	CID_70128847	7.46	N
83	CID_70128902	6.50	Y
84	CID_70128904	6.57	Y
85	CID_70128987	5.60	Y
86	CID_70129065	6.69	N
87	CID_70129161	7.05	Y
88	CID_70129198	6.53	Y
89	CID_70129204	7.14	N
90	CID_70129375	6.18	Y
91	CID_70129377	6.74	Y
92	CID_70129380	7.12	Y
93	CID_9804916	6.38	Y
94	CID_9826776	6.33	Y
95	CID_9827285	6.48	Y
96	CID_9828392	6.12	Y
97	CID_9828587	6.86	N
98	CID_9829426	6.45	Y
99	CID_9891033	6.14	Y
100	CID_9893352	5.94	Y
101	CID_9933226	6.34	Y
102	CID_9935104	6.56	Y
103	CID_9935196	6.57	Y
104	CID_9935788	6.50	Y
105	CID_9936347	6.45	Y
106	CID_9936803	6.36	Y
107	CID_9955874	6.20	Y
108	CID_9957122	6.62	Y
109	CID_9957692	6.57	Y
110	CID_9958161	6.19	Y

^a^AD: model structural applicability domain; Y: compound inside the model structural AD; N: compound outside the model structural AD.

**Table 5:** The calculated binding free energies (kcal mol^−1^) of four systems.

Complex	Contribution								
	Δ*E*_ele_	Δ*E*_vdw_	Δ*G*_p_	Δ*G*_np_	Δ*E*_MM_	Δ*G*_sol_	Δ*E*_bind_	−*T*Δ*S*	Δ*G*_bind_
Compound 4	−29.58	−58.93	44.95	−6.74	−88.50	38.22	−50.28	27.59	−22.69
CID_70128824	−3.32	−70.56	24.14	−7.25	−73.88	16.89	−56.99	25.13	−31.86
CID_70127147	−16.79	−71.34	33.50	−7.80	−88.13	25.69	−62.44	29.38	−33.06
CID_70126881	−10.01	−69.32	25.14	−8.85	−79.33	16.29	−63.05	21.43	−41.62
